# Supplementary material for: Multi-omics analysis of aggregative multicellularity
Source: iScience. 2024 Aug 3;27(9):110659. doi: 10.1016/j.isci.2024.110659 (PMC11367525; doi:10.1016/j.isci.2024.110659)
Supplement: Document S1. Figures S1–S10 [file mmc1.pdf]

iScience, Volume *27*

Supplemental information

Multi-omics analysis of aggregative multicellularity

Bart Edelbroek, Jakub Orzechowski Westholm, Jonas Bergquist, and Fredrik Söderbom

Supplemental Figures

Spearman correlation between mRNA samples

	0h_a	0h_b	0h_c	0h_d	2h_a	2h_b	2h_c	2h_d	4h_a	4h_b	4h_c	4h_d	6h_a	6h_b	6h_c	6h_d	8h_a	8h_b	8h_c	8h_d	10h_a	10h_b	10h_c	10h_d
0h_a	1.00	0.99	0.98	0.97	0.90	0.91	0.89	0.90	0.89	0.89	0.86	0.90	0.83	0.86	0.88	0.83	0.69	0.76	0.75	0.73	0.64	0.73	0.71	0.67
0h_b	0.99	1.00	0.99	0.99	0.90	0.92	0.87	0.90	0.88	0.88	0.84	0.90	0.82	0.84	0.88	0.82	0.68	0.78	0.74	0.73	0.66	0.72	0.72	0.68
0h_c	0.98	0.99	1.00	1.00	0.89	0.91	0.87	0.89	0.88	0.88	0.85	0.90	0.83	0.85	0.89	0.82	0.69	0.78	0.75	0.74	0.66	0.73	0.73	0.69
0h_d	0.97	0.99	1.00	1.00	0.88	0.90	0.86	0.89	0.88	0.89	0.85	0.90	0.83	0.85	0.89	0.83	0.69	0.78	0.75	0.74	0.67	0.74	0.74	0.69
2h_a	0.90	0.90	0.89	0.88	1.00	0.99	0.95	0.96	0.92	0.91	0.89	0.93	0.87	0.88	0.89	0.86	0.75	0.80	0.78	0.78	0.69	0.77	0.75	0.71
2h_b	0.91	0.92	0.91	0.90	0.99	1.00	0.94	0.97	0.93	0.92	0.89	0.94	0.87	0.87	0.90	0.86	0.74	0.82	0.78	0.78	0.70	0.77	0.76	0.73
2h_c	0.89	0.87	0.87	0.86	0.95	0.94	1.00	0.94	0.93	0.92	0.92	0.93	0.88	0.89	0.89	0.85	0.76	0.81	0.84	0.81	0.70	0.77	0.76	0.72
2h_d	0.90	0.90	0.89	0.89	0.96	0.97	0.94	1.00	0.92	0.91	0.87	0.95	0.87	0.85	0.91	0.85	0.74	0.85	0.81	0.81	0.74	0.77	0.80	0.77
4h_a	0.89	0.88	0.88	0.88	0.92	0.93	0.93	0.92	1.00	0.99	0.96	0.98	0.97	0.95	0.97	0.95	0.84	0.88	0.87	0.86	0.76	0.84	0.81	0.78
4h_b	0.89	0.88	0.88	0.89	0.91	0.92	0.92	0.91	0.99	1.00	0.95	0.96	0.96	0.96	0.97	0.95	0.82	0.86	0.85	0.84	0.75	0.83	0.79	0.76
4h_c	0.86	0.84	0.85	0.85	0.89	0.89	0.92	0.87	0.96	0.95	1.00	0.96	0.94	0.96	0.94	0.91	0.83	0.83	0.87	0.84	0.72	0.82	0.78	0.74
4h_d	0.90	0.90	0.90	0.90	0.93	0.94	0.93	0.95	0.98	0.96	0.96	1.00	0.95	0.94	0.97	0.92	0.82	0.89	0.88	0.87	0.77	0.83	0.83	0.79
6h_a	0.83	0.82	0.83	0.83	0.87	0.87	0.88	0.87	0.97	0.96	0.94	0.95	1.00	0.97	0.98	0.98	0.91	0.92	0.92	0.92	0.81	0.89	0.84	0.81
6h_b	0.86	0.84	0.85	0.85	0.88	0.87	0.89	0.85	0.95	0.96	0.96	0.94	0.97	1.00	0.97	0.96	0.86	0.85	0.88	0.86	0.74	0.87	0.79	0.76
6h_c	0.88	0.88	0.89	0.89	0.89	0.90	0.89	0.91	0.97	0.97	0.94	0.97	0.98	0.97	1.00	0.97	0.86	0.91	0.90	0.89	0.79	0.87	0.84	0.81
6h_d	0.83	0.82	0.82	0.83	0.86	0.86	0.85	0.85	0.95	0.95	0.91	0.92	0.98	0.96	0.97	1.00	0.90	0.90	0.89	0.90	0.79	0.89	0.82	0.81
8h_a	0.69	0.68	0.69	0.69	0.75	0.74	0.76	0.74	0.84	0.82	0.83	0.82	0.91	0.86	0.86	0.90	1.00	0.93	0.95	0.97	0.91	0.96	0.91	0.90
8h_b	0.76	0.78	0.78	0.78	0.80	0.82	0.81	0.85	0.88	0.86	0.83	0.89	0.92	0.85	0.91	0.90	0.93	1.00	0.95	0.97	0.92	0.92	0.94	0.92
8h_c	0.75	0.74	0.75	0.75	0.78	0.78	0.84	0.81	0.87	0.85	0.87	0.88	0.92	0.88	0.90	0.89	0.95	0.95	1.00	0.98	0.89	0.92	0.91	0.89
8h_d	0.73	0.73	0.74	0.74	0.78	0.78	0.81	0.81	0.86	0.84	0.84	0.87	0.92	0.86	0.89	0.90	0.97	0.97	0.98	1.00	0.92	0.95	0.94	0.93
10h_a	0.64	0.66	0.66	0.67	0.69	0.70	0.70	0.74	0.76	0.75	0.72	0.77	0.81	0.74	0.79	0.79	0.91	0.92	0.89	0.92	1.00	0.93	0.97	0.98
10h_b	0.73	0.72	0.73	0.74	0.77	0.77	0.77	0.77	0.84	0.83	0.82	0.83	0.89	0.87	0.87	0.89	0.96	0.92	0.92	0.95	0.93	1.00	0.95	0.94
10h_c	0.71	0.72	0.73	0.74	0.75	0.76	0.76	0.80	0.81	0.79	0.78	0.83	0.84	0.79	0.84	0.82	0.91	0.94	0.91	0.94	0.97	0.95	1.00	0.98
10h_d	0.67	0.68	0.69	0.69	0.71	0.73	0.72	0.77	0.78	0.76	0.74	0.79	0.81	0.76	0.81	0.81	0.90	0.92	0.89	0.93	0.98	0.94	0.98	1.00

Spearman correlation between protein samples

	0h_a	0h_b	0h_c	2h_a	2h_b	2h_c	4h_a	4h_b	4h_c	8h_a	8h_b	8h_c	10h_a	10h_b	10h_c
0h_a	1.00	0.96	0.95	0.95	0.95	0.92	0.93	0.94	0.94	0.87	0.89	0.90	0.82	0.87	0.86
0h_b	0.96	1.00	0.96	0.95	0.96	0.93	0.93	0.95	0.94	0.87	0.88	0.89	0.82	0.87	0.87
0h_c	0.95	0.96	1.00	0.94	0.95	0.92	0.92	0.94	0.94	0.87	0.88	0.89	0.82	0.87	0.87
2h_a	0.95	0.95	0.94	1.00	0.97	0.94	0.95	0.96	0.96	0.89	0.91	0.91	0.84	0.89	0.88
2h_b	0.95	0.96	0.95	0.97	1.00	0.94	0.96	0.97	0.97	0.90	0.91	0.92	0.84	0.89	0.89
2h_c	0.92	0.93	0.92	0.94	0.94	1.00	0.94	0.94	0.94	0.90	0.91	0.92	0.85	0.89	0.90
4h_a	0.93	0.93	0.92	0.95	0.96	0.94	1.00	0.96	0.96	0.92	0.93	0.95	0.86	0.91	0.91
4h_b	0.94	0.95	0.94	0.96	0.97	0.94	0.96	1.00	0.98	0.91	0.92	0.94	0.86	0.91	0.91
4h_c	0.94	0.94	0.94	0.96	0.97	0.94	0.96	0.98	1.00	0.90	0.92	0.94	0.85	0.90	0.90
8h_a	0.87	0.87	0.87	0.89	0.90	0.90	0.92	0.91	0.90	1.00	0.96	0.96	0.94	0.96	0.96
8h_b	0.89	0.88	0.88	0.91	0.91	0.91	0.93	0.92	0.92	0.96	1.00	0.96	0.93	0.95	0.94
8h_c	0.90	0.89	0.89	0.91	0.92	0.92	0.95	0.94	0.94	0.96	0.96	1.00	0.92	0.96	0.96
10h_a	0.82	0.82	0.82	0.84	0.84	0.85	0.86	0.86	0.85	0.94	0.93	0.92	1.00	0.94	0.95
10h_b	0.87	0.87	0.87	0.89	0.89	0.89	0.91	0.91	0.90	0.96	0.95	0.96	0.94	1.00	0.96
10h_c	0.86	0.87	0.87	0.88	0.89	0.90	0.91	0.91	0.90	0.96	0.94	0.96	0.95	0.96	1.00

Figure S1. Correlation between biological replicates of transcriptomics and proteomics, related to Figure 2. Correlation matrix of all transcriptomics samples (top) and proteomics samples (bottom), with the Spearman correlation indicated for each comparison. Biological replicates of the same time point are indicated with a black box.

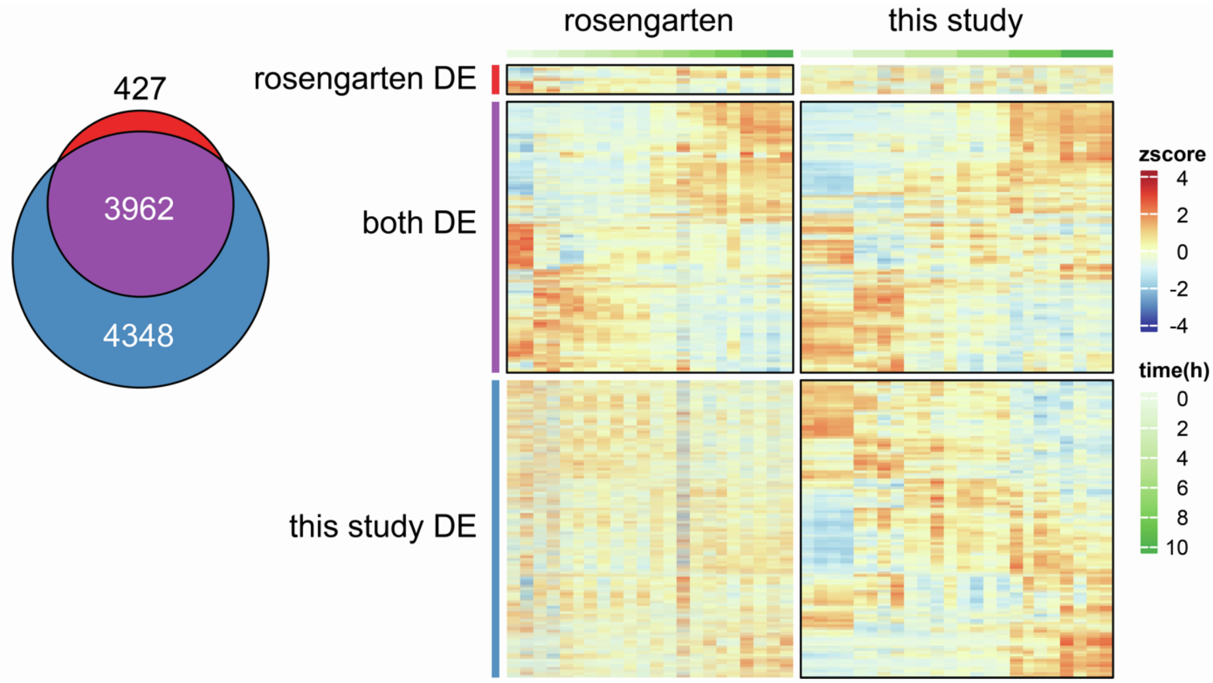

Figure S2. Regulated mRNA compared to Rosengarten et al, related to Figure 2. Comparison of the transcriptomics dataset generated in this study (this study) with the 0h to 10h time points of the dataset generated by Rosengarten et al.[1] (rosengarten). Red and purple: 4389 protein coding transcripts identified as differentially expressed in the Rosengarten dataset; blue and purple: 8310 protein coding transcripts in the dataset generated in this study; purple: 3962 protein coding transcripts identified in both studies. Regulation of the transcripts is shown by z-score from 0h growing cells to 10h post initiation of development, with differentially expressed transcripts outlined (black rectangles).

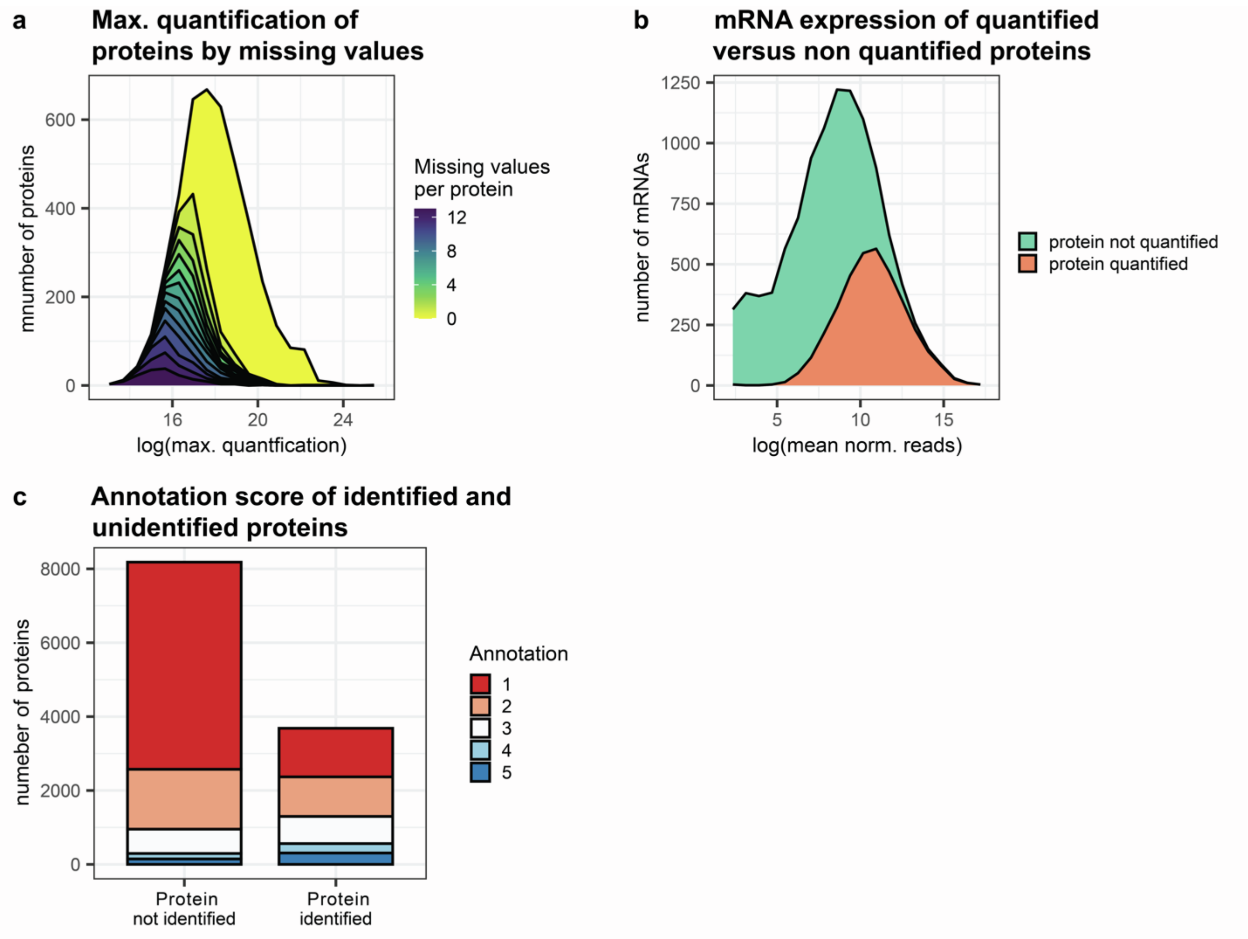

Figure S3. Analysis of missing proteins, related to Figure 3a. a Protein quantification grouped by the number of missing values. For each protein, the maximum value among all samples was calculated. Proteins without missing values across all samples are yellow. For proteins with all missing values, no maximum value could be calculated and these are omitted. **b** mRNA expression in log-scaled normalized reads. Reads were averaged across biological replicates and time points for each gene, classified by whether or not the cognate protein was quantified from the proteomics analysis. **c** Annotation score of not identified proteins, and identified proteins, ranging from 1 (lowest annotation score) to 5 (maximum annotation score), accessed from UniProt[2].

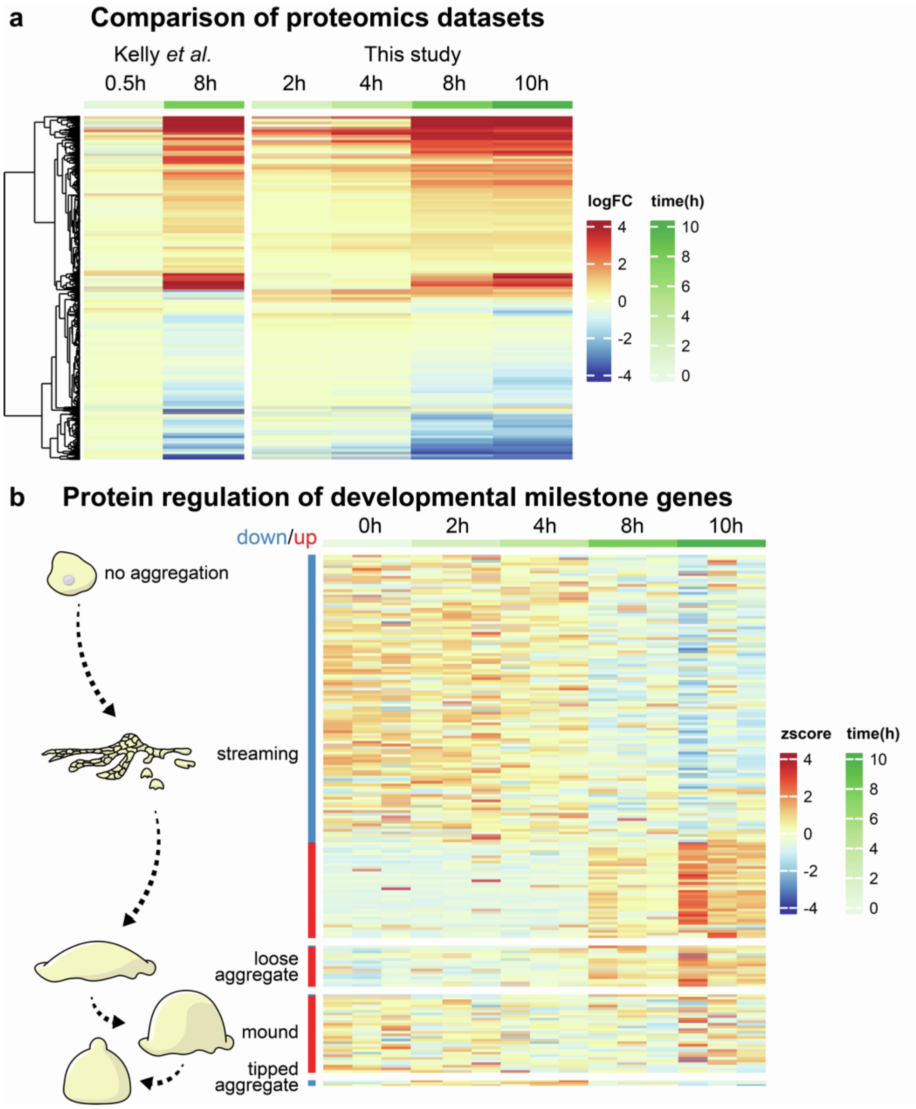

Figure S4. Protein regulation over time compared to previous data and for milestone genes, related to Figure 3. a Regulation of proteins over time in our dataset (This study) compared to the dataset by Kelly et al., accessed through the ProteomeXchange[3,4]. Regulation in log fold change (logFC) of time points relative to non-developed cells. Proteins included in heatmap were differentially expressed in either dataset. Proteins with missing values in either dataset, were omitted. **b** Regulation of the milestone gene proteins over time. Milestone genes are included which characterize the transition from "no aggregation" to "streaming" (streaming), from "streaming" to "loose aggregate" (loose aggregate), from "loose aggregate" to "mound" (mound) and from "mound" to "tipped aggregate"[5], from top to bottom. Illustrations of the morphological structures are included on the left. Whether the milestone genes are defined as downregulated (blue) or upregulated (red) in the transition, is annotated on the left. Only genes are included for which protein could be quantified across all timepoints and replicates.

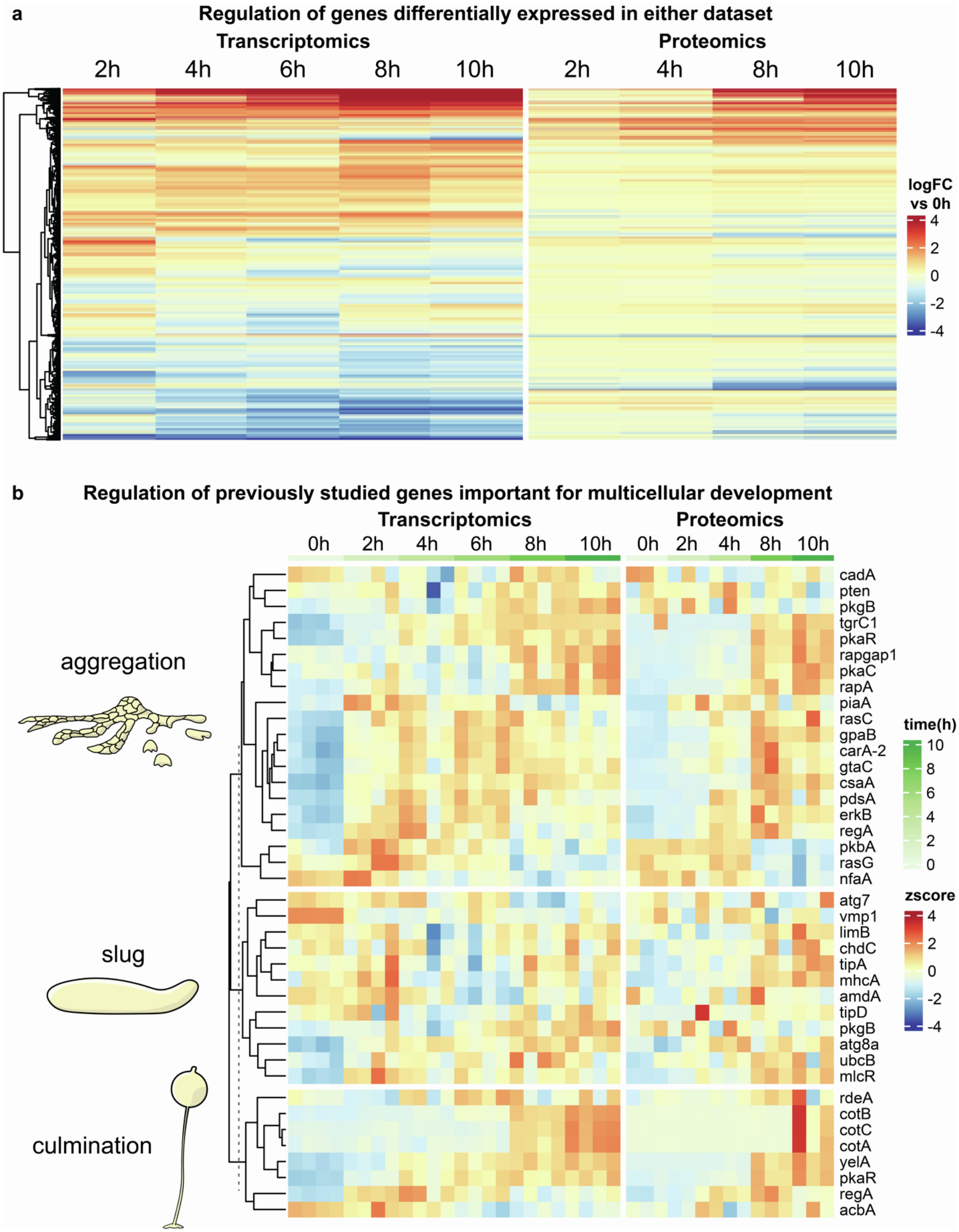

Figure S5. Regulation of genes in both transcriptomics and proteomics datasets, related to Figure 4. a Regulation of all genes that are differentially expressed in either dataset, and contained no missing values (2934 genes). Log fold change (logFC) is calculated for the indicated time points versus the 0h time

point. The genes in the heatmap are hierarchically clustered based on their regulation, with the dendrogram shown left. **b** Transcript and protein expressed for genes involved in regulation of aggregation, slug and culmination (fruiting body) multicellular stages, as presented by Loomis (2015)[6].

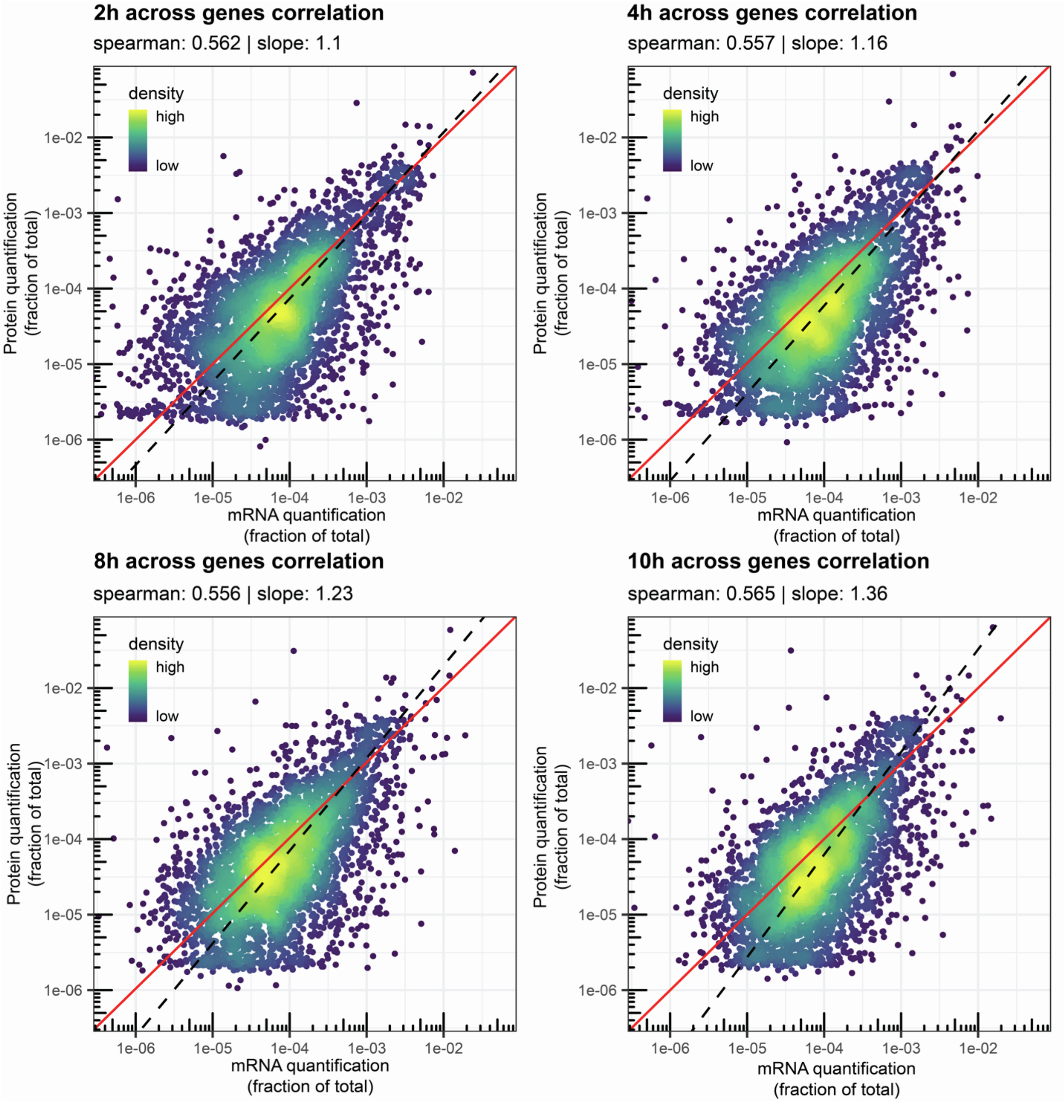

Figure S6. Across genes correlation of mRNA and protein fractions at 2h, 4h, 8h, and 10h time points, related to Figure 4b. Correlation of the mean mRNA and protein levels across the indicated time point. Each dot represents the mean protein and mRNA expression from a single gene. The dashed black line indicates the linear regression of the data, with the slope indicated above the plot. The red line is the y=x diagonal.

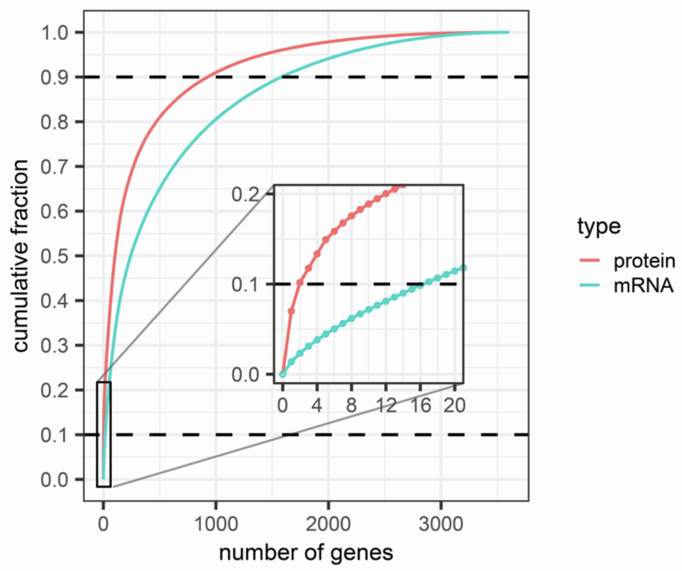

	Number of proteins	Number of mRNAs
Top 10%	2	16
Bottom 10%	2679	2023

Figure S7. Differences in dynamic range between transcriptomics and proteomics, related to Figure 4. Cumulative fraction of proteomics and transcriptomics summing up to 1, for all genes quantified in both datasets. The dashed lines indicate the top and bottom 10% of expression, and the number of genes included in these cutoffs for the different datasets is included in the table.

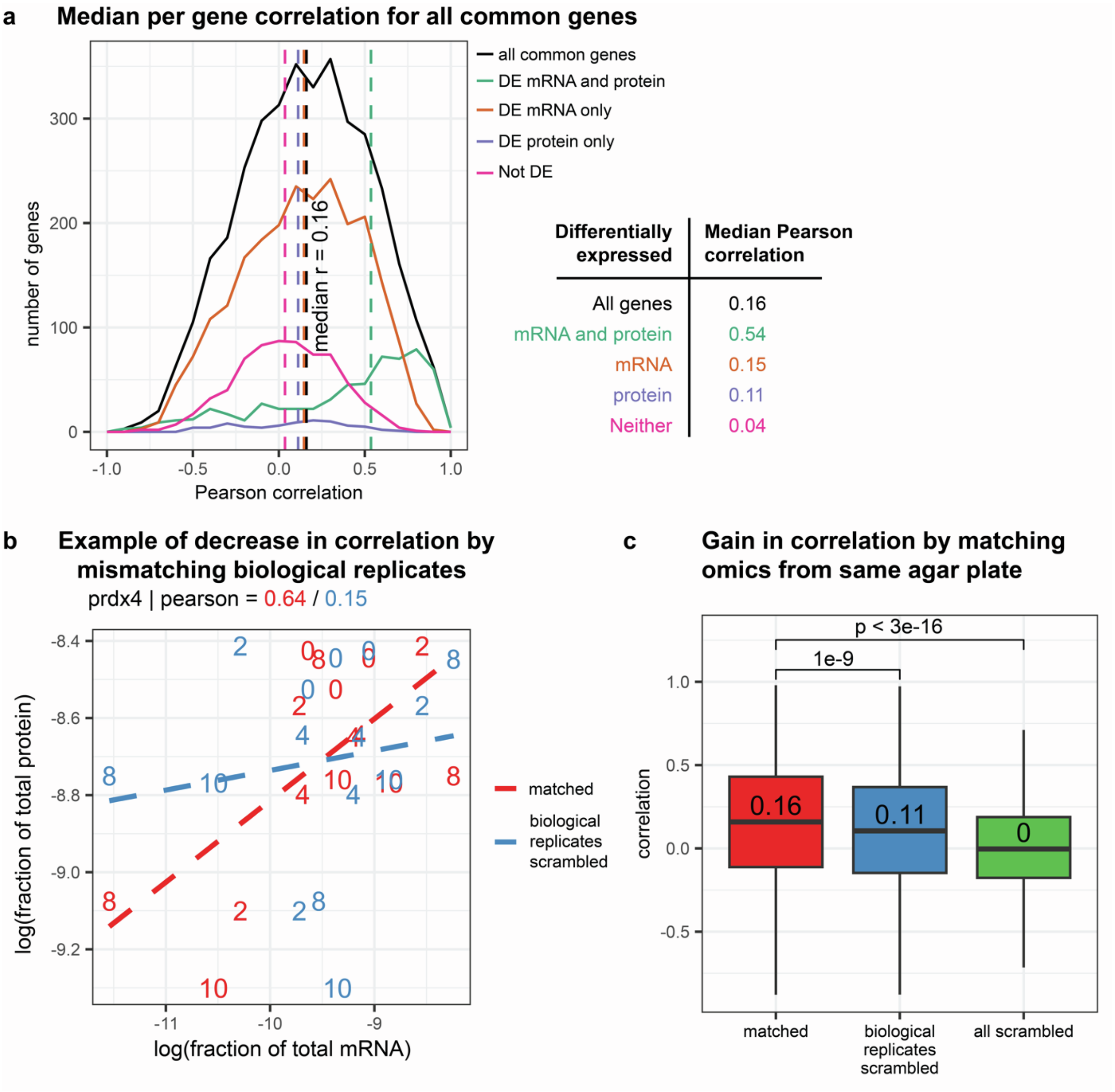

Figure S8. Per gene mRNA and protein quantification, related to Figure 4e. a Distribution of per gene Pearson correlations for all common genes in the transcriptomics and proteomics datasets, and genes grouped by differentially expression in either of the datasets. The median Pearson correlation for each group of genes is visualized with a dashed line, and is noted in the table on the right. **b** Difference in Pearson correlation for prdx4 gene when mismatching biological replicates. Each replicate is plotted based on the mRNA abundance and protein abundance of either that same replicate (red) or protein abundance of another replicate from the same time point (blue). Linear regression indicated with dashed lines matching the color of the replicates, with a Pearson correlation indicated above the plot. **c** Boxplots of per gene correlation for all genes in the proteomics and transcriptomics datasets, either where the data is matched to the same biological replicate from the same plate (red), or where the data is matched to different biological replicates from the same time point (blue), or where the data is completely scrambled, i.e. protein data from one biological replicate and time point, matched to mRNA data from another biological replicate and time point (green). P-values from two-tailed t-test for indicated pairwise comparisons.

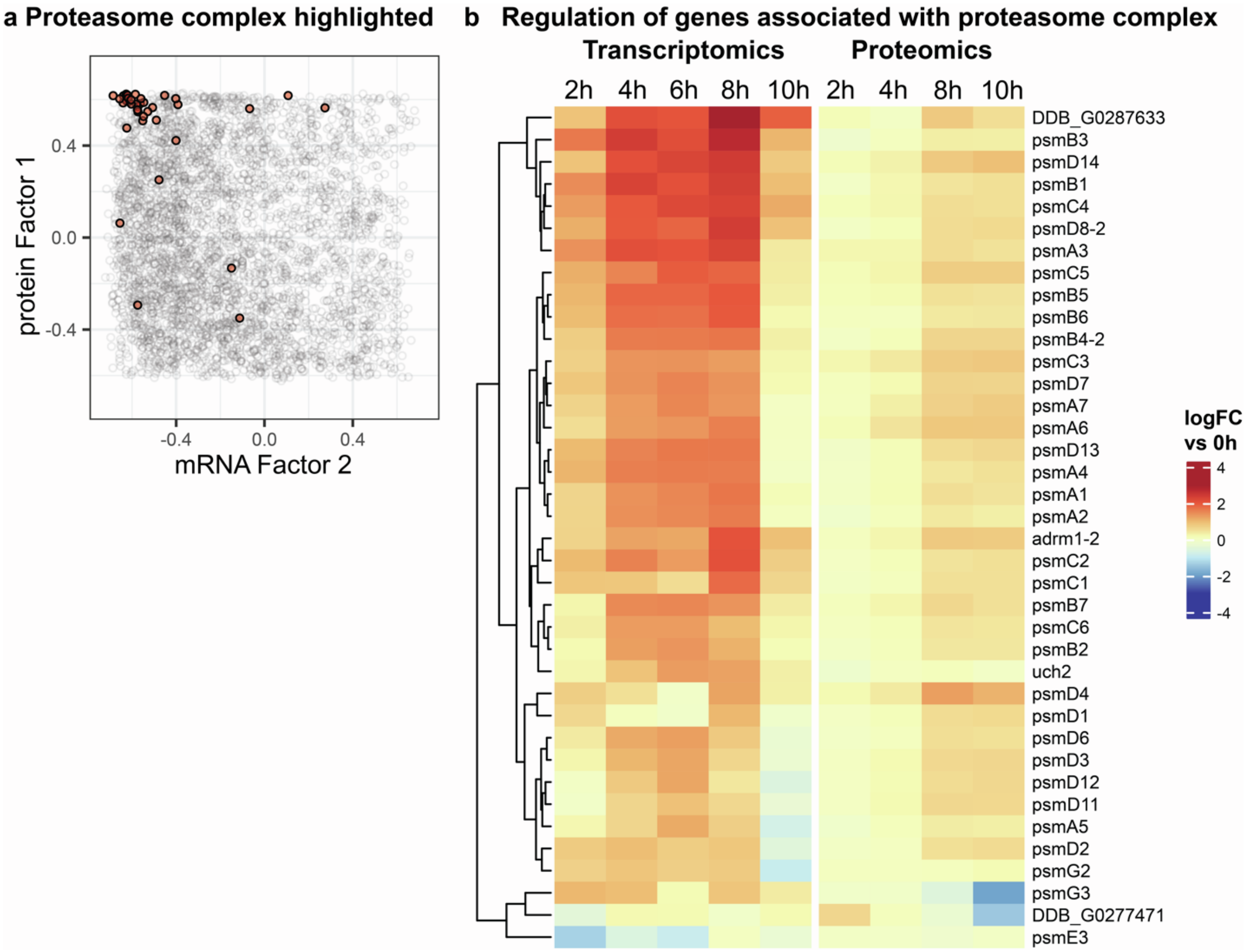

Figure S9. Regulation of the proteasome complex during development, related to Figure 5. a Highlight of members of the proteasome complex in mRNA factor 2 by protein factor 1 plot. In light-grey the distribution of all genes is shown. Genes in the top-left are associated with high Factor 1 at the protein modality, and low Factor 2 at the mRNA modality **b** Regulation of proteasome complex genes in transcriptomics and proteomics datasets in log fold change (logFC) versus the 0h time point.

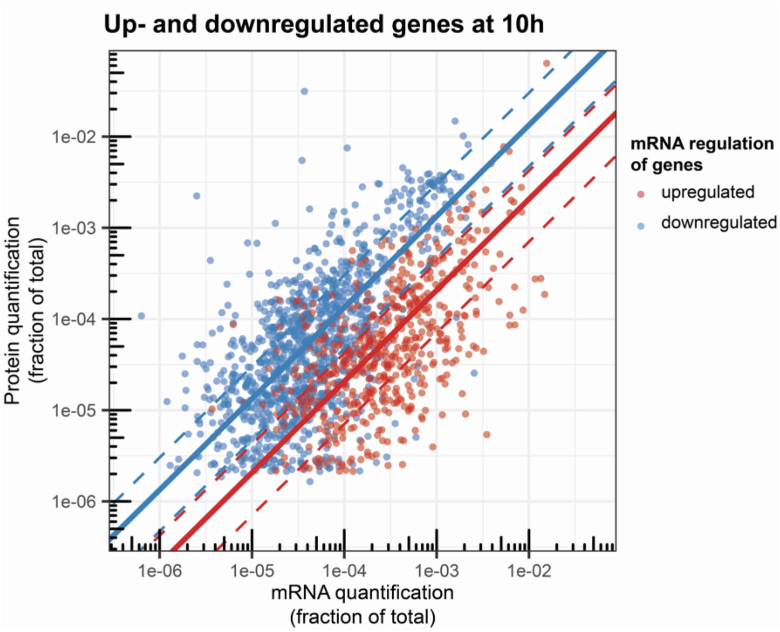

Figure S10. Fraction of protein to mRNA for the 10h time point, related to Figure 6d, e. Across genes correlation of genes quantified in the transcriptomics and proteomics datasets. Each gene is plotted by the mean mRNA level and mean protein level at the 10h time point. Genes are included for which the mRNA is upregulated at the 10h time point (red) or genes for which the mRNA is downregulated at the 10h time point (blue). The median protein to mRNA ratio for each set of genes is indicated with the solid line in matching color. The 25th and 75th percentile are indicated with dashed lines.

Supplemental References

1. Rosengarten, R.D., Santhanam, B., Fuller, D., Katoh-Kurasawa, M., Loomis, W.F., Zupan, B., and Shaulsky, G. (2015). Leaps and lulls in the developmental transcriptome of Dictyostelium discoideum. BMC Genomics *16*, 294. https://doi.org/10.1186/s12864-015-1491-7.

2. The UniProt Consortium (2023). UniProt: the Universal Protein Knowledgebase in 2023. Nucleic Acids Research *51*, D523–D531. https://doi.org/10.1093/nar/gkac1052.

3. Kelly, B., Carrizo, G.E., Edwards-Hicks, J., Sanin, D.E., Stanczak, M.A., Priesnitz, C., Flachsmann, L.J., Curtis, J.D., Mittler, G., Musa, Y., et al. (2021). Sulfur sequestration promotes multicellularity during nutrient limitation. Nature *591*, 471–476. https://doi.org/10.1038/s41586-021-03270-3.

4. ProteomeXchange Dataset PXD023404 https://proteomecentral.proteomexchange.org/cgi/GetDataset?ID=PXD023404.

5. Katoh-Kurasawa, M., Hrovatin, K., Hirose, S., Webb, A., Ho, H.-I., Zupan, B., and Shaulsky, G. (2021). Transcriptional milestones in Dictyostelium development. Genome Res *31*, 1498–1511. https://doi.org/10.1101/gr.275496.121.

6. Loomis, W.F. (2015). Genetic control of morphogenesis in *Dictyostelium*. Developmental Biology *402*, 146–161. https://doi.org/10.1016/j.ydbio.2015.03.016.
